# Novel Techniques with the Aid of a Staged CBCT Guided Surgical Protocol

**DOI:** 10.1155/2015/439706

**Published:** 2015-01-06

**Authors:** Evdokia Chasioti, Mohammed Sayed, Howard Drew

**Affiliations:** ^1^Department of Periodontics, Rutgers School of Dental Medicine, Rutgers University, 110 Bergen Street, Newark, NJ 07101, USA; ^2^Department of Restorative Dentistry, Rutgers School of Dental Medicine, Rutgers University, 110 Bergen Street, Newark, NJ 07101, USA

## Abstract

The case report will present some novel techniques for using a “staged” protocol utilizing strategic periodontally involved teeth as transitional abutments in combination with CBCT guided implant surgery. Staging the case prevented premature loading of the grafted sites during the healing phase. A CBCT following a tenting screw guided bone regeneration procedure ensured adequate bone to place an implant fixture. Proper assessment of the CBCT allowed the surgeon to do an osteotome internal sinus lift in an optimum location. The depth of the bone needed for the osteotome sinus floor elevation was planned. The staged appliance allowed these sinus-augmented sites to heal for an extended period of time compared to implants, which were uncovered and loaded at an earlier time frame. The staged protocol and CBCT analysis enabled the immediate implants to be placed in proper alignment to the adjacent fixture. After teeth were extracted, the osseointegrated implants were converted to abutments for the transitional appliance. Finally, the staged protocol allowed for soft tissue enhancement in the implant and pontic areas prior to final insertion of the prosthesis.

## 1. Introduction

Almost all success criteria in implant dentistry rely on accurate implant placement based on the presurgical treatment plan [1, 2]. In the past, several diagnostic tools and techniques have been used for implant placement. Among those diagnostic tools are conventional dental radiographs that are used to estimate the amount of available bone for implant placement [3]. Panoramic radiographs which are 2-dimensional have a magnification error of 24% horizontally and 36% vertically [4, 5]. Arbitrary methods for fabrication of surgical guides on diagnostic cast have been proposed. A simplified technique for surgical guide fabrication does not take into account the thickness of soft tissue and the location of anatomical structures relative to proposed implant sites [6]. In modern dentistry, the use of advanced technology in diagnosis, treatment planning, and fabrication of various appliances and prosthetics has taken the field to an entirely different level. CBCT scan imaging allows the dentist to evaluate craniofacial structures in three dimensions with acceptable accuracy, thus reducing the magnification and arbitrary planning in implant dentistry [7].

This paper discusses a staged protocol that can make difficult cases more manageable, based on a three-dimensional treatment plan, with novel surgical techniques and fixed provisional restorations. Modified mucosal-teeth supported radiographic and surgical guides allowed for bilateral sinus-floor elevations and simultaneous implant placement. Additionally, the benefits of staging the case until the final restoration is delivered are presented.

## 2. Materials and Methods

**Figure 1:**
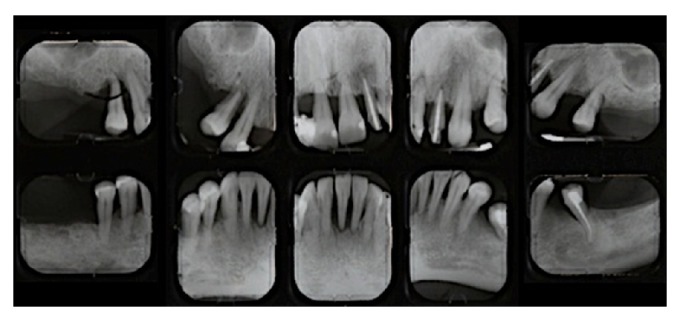
Pre-treatment periapical radiographs.

A 47-year-old Caucasian woman with a nonsignificant medical history presented with generalized chronic severe periodontitis with secondary occlusal trauma (Figure 1).

**Figure 2:**
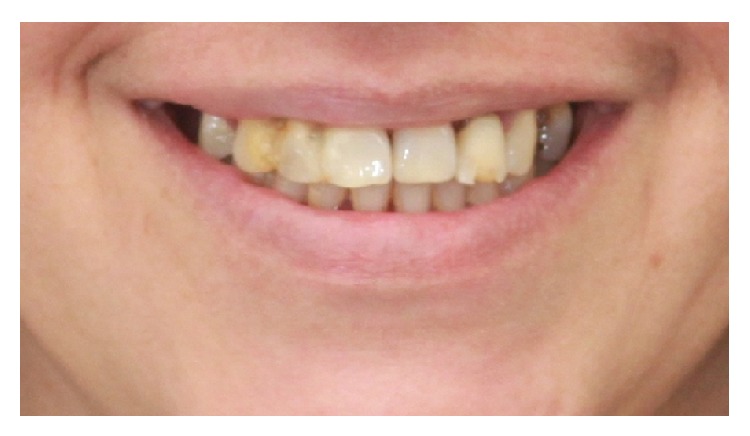
Pre-treatment smile photograph.

Primary etiologic factors included heavy accumulation of plaque and calculus, with secondary factors including defective restorations and occlusal trauma. She presented with cyanotic edematous gingiva, severe clinical attachment loss, and mucogingival defects and recession. Upon occlusal evaluation, it was revealed that the maxillary midline coincided with the facial midline, with horizontal overlap of 1 mm and vertical overlap of 2 mm. Smile analysis revealed that the patient had an average smile line and maxillary incisal edges did not follow the curvature of the lower lip (Figure 2). Centric occlusion to maximum intercuspation discrepancy was found to be 1 mm forward.

**Figure 3:**
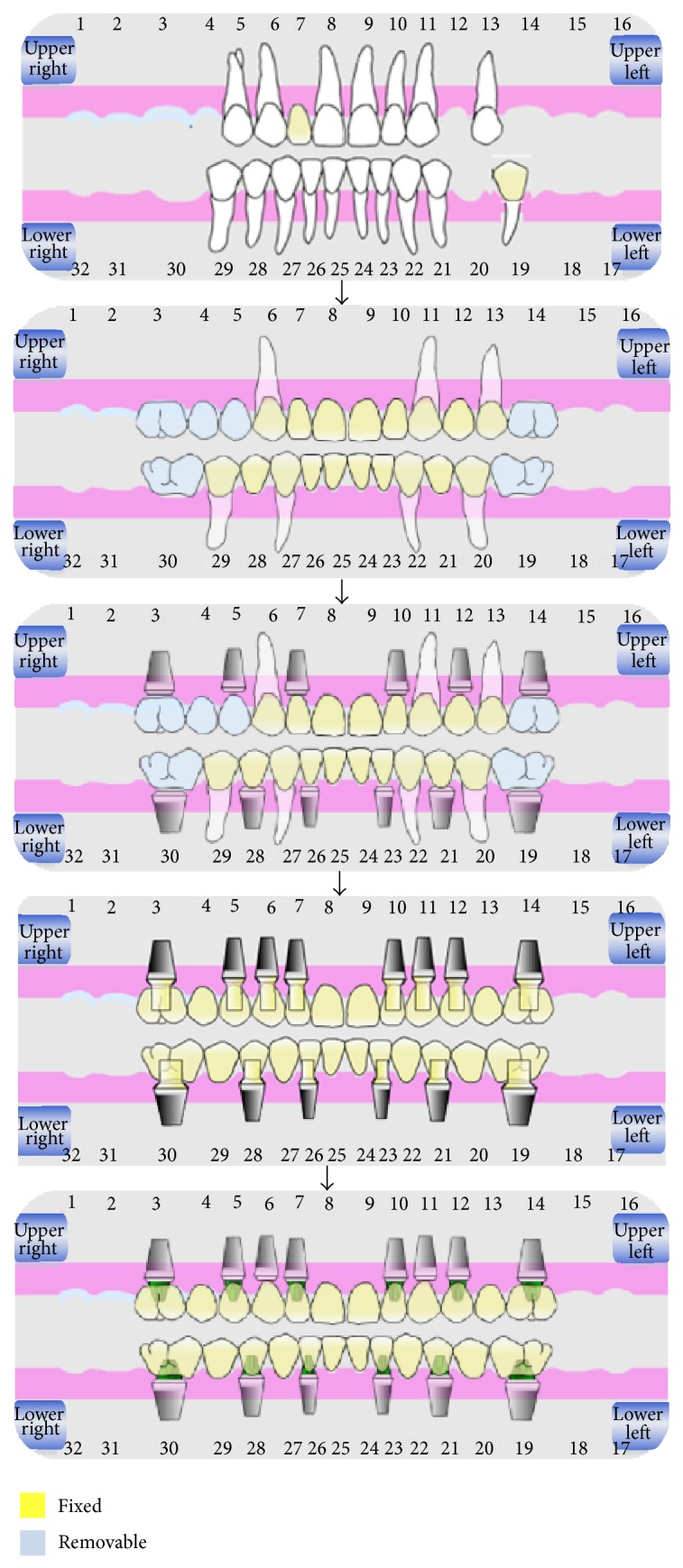
Schematic showing the steps of staged approach treatment plan.

The patients' final treatment plan included full mouth rehabilitation through a staged approach with guided implant surgery. The final implant abutments were selected on the guided software in areas # 3, 5, 7, 10, 12, and 14 for the maxilla and 19, 21, 23, 26, 28, and 30 for the mandible. The case was treatment-planned to be staged using selected maxillary teeth # 6, 11, and 13 and the mandibular teeth # 20, 22, 27, and 29 to be retained as abutments for a fixed temporary prosthesis with a transitional removable prosthesis (Figure 3).

The first phase of the periodontal treatment plan included oral hygiene instruction and full mouth supragingival debridement, followed by root planning with subgingival irrigation with chlorhexidine gluconate on the temporary abutment teeth. The criteria for the retention of these teeth included decreased mobility and probing depths and a favorable prognosis compared to the remaining hopeless teeth. Selected teeth were prepared and single unit provisional crowns were fabricated using temporary acrylic material (Alike, GC America Inc., Alsip, IL) and cemented with temporary cement (Tempbond NE, Kerr Manufacturing Co., Romulus, MI). At the re-evaluation visit, the patient presented with significantly improved oral hygiene and a reduced plaque index.

**Figure 4:**
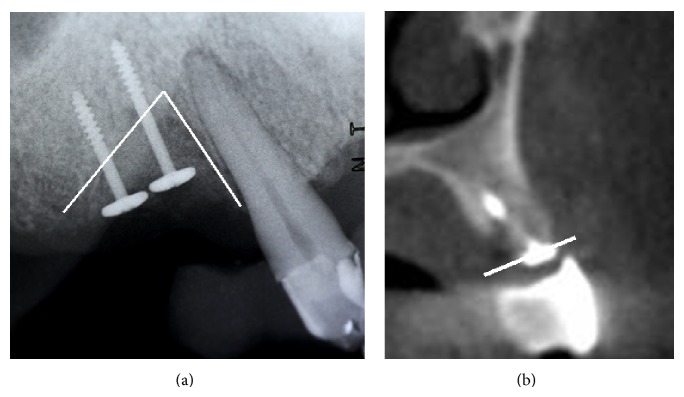
(a) Tenting screw technology for implant # 5 site development. (b) Significant bone increase in vertical and horizontal dimensions, confirmed by the CBCT sagittal slice.

Upon phase 1 re-evaluation, the first surgical phase of the treatment included extraction of hopeless maxillary and mandibular teeth, utilizing GBR with mineralized crushed cortical bone (RegenerOss Allograft, Biomet 3i) and a resorbable collagen membrane (OsseoGuard Flex, Biomet 3i) for the deficient extraction socket areas, # 3, 7 to 10, 23 to 26, 21, and 28. Tooth # 5 was selected for extraction and GBR with tenting screw technology [8] as part of the maxillary grafting procedures (Figure 4).

**Figure 5:**
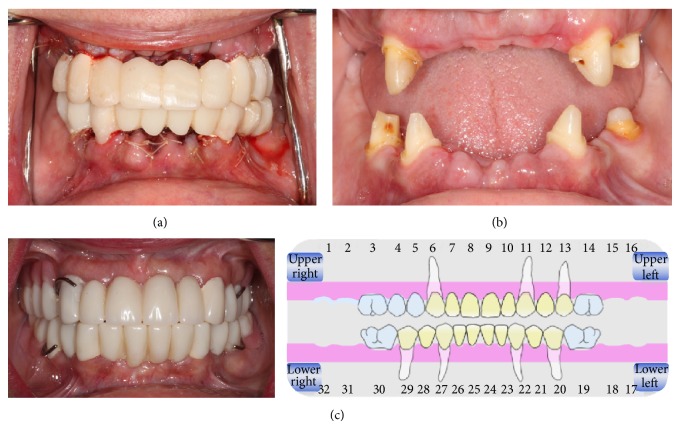
Steps of phase I treatment. (a) First set of provisional prostheses delivered after planned teeth extraction. (b) Intraoral picture 1 month after planned teeth extraction. (c) Intraoral picture depicting delivery of enhanced provisional prostheses.

Periosteal releasing incisions were followed by horizontal mattress and single interrupted Vicryl 4.0 sutures, stabilizing the flap and achieving primary closure. The patient was prescribed amoxicillin 500 mg every eight hours for 7 days, chlorhexidine gluconate rinse twice a day for 2 weeks, nonsteroidal anti-inflammatory medication (ibuprofen 600 mg, q4–6 h) for 7 days postoperatively, and 2 grams of amoxicillin premedication. Preparation for full coverage restoration was finalized and preliminary alginate impressions (Jeltrate Plus, Dentsply Caulk, York, PA) were made to create diagnostic casts. Record bases with occlusal rims were fabricated on the diagnostic casts, which were mounted on semiadjustable articulator (Hanau Wide Vue, Whip Mix, Fort Collins, CO) using facebow transfer and centric relation records. Intraoral protrusive record was made to program the horizontal condylar inclination. The lateral condylar inclination was calculated based on Hanau formula (L = H/8 + 12). The result of the formula was used to program the lateral condylar inclination on the articulator. Full mouth diagnostic setup using denture teeth was completed on the mounted casts. The casts with diagnostic setup were duplicated and provisional fixed prostheses were then fabricated and cemented on abutments teeth # 6, 11, and 13 with a cantilever pontic # 5 for the maxillary arch. The initial set of temporary prostheses was delivered immediately after extraction of the hopeless maxillary and mandibular teeth. It was used as a template for adjustments in order to arrive at optimized esthetics, phonetics, and function before fabrication of metal-reinforced laboratory processed provisional prosthesis. Bilateral internal sinus lifts in the areas # 3 and 14 and implant placement were scheduled in 3 months [9]. Abutment teeth # 20, 22, 27, and 29 were used to retain a mandibular fixed provisional prosthesis (Figure 5).

After 3 months of uneventful healing, another set of provisional fixed prostheses for improved esthetics, phonetics, function, and strength were fabricated. Alginate impressions were made for the previously optimized provisional prostheses and prepared teeth. Alginate impressions were poured in dental stone. The casts of the maxillary and mandibular prepared teeth were cross-mounted to the casts of the optimized provisional prosthesis. Denture teeth were set on the casts of prepared teeth, which were hollowed out from the palatal/lingual aspect to accommodate the prepared teeth and create space for the metal frames that were cast with beads to retain denture teeth and temporary acrylic material (Alike, GC America Inc., Alsip, IL). The metal-reinforced provisional prostheses were relined with acrylic material and cemented over abutment teeth using temporary cement (Tempbond NE, Kerr Manufacturing Co., Romulus, MI). Interim removable partial dentures were delivered to replace the missing posterior teeth for better esthetics and function.

The provisional fixed prostheses were evaluated and adjusted periodically, if needed, to enhance esthetics and phonetics. Alginate impressions were made from the provisional fixed prostheses, which were then removed, and another set of alginate impressions were made of the prepared abutment teeth. The casts were then cross-mounted on a semiadjustable articulator. Mucosa-tooth supported radiographic guides were fabricated over the prepared teeth using orthodontic resin (Dentsply International Inc., Milford, DE) mixed with barium sulfate for the teeth and orthodontic resin only for the denture base. Straumann templiX reference plates, containing three titanium reference pins, were attached to the radiographic guides where Straumann gonyX was used to record reference pins orientation.

The second surgical phase initiated with CBCT evaluation and treatment planning of restoratively dictated implant positions. The reference pins were detected on the coDiagnostiX software. The match between the software planning and the reference pins on the radiographic guides was ensured. The radiographic guides were fitted back on the casts of prepared teeth. The casts were remounted on Straumann gonyX using previous reference pins orientation. The radiographic guides were then converted to surgical guides with addition of metal sleeves based on spatial position and depth information provided by coDiagnostiX software. A flapless approach and CBCT guided surgery with tooth-stabilized mucosa-supported surgical guides for minimally invasive intervention were the treatment of choice. Two-staged bone level SLActive titanium implants were placed at # 3, 5, 7, 10, and 14 sites in the maxilla and at # 19, 21, 23, 26, 28, and 30 positions in the mandible. The canine positions in the mandible were not considered as potential implant sites because there was enough bone quantity at the lateral incisors position, which provided proper implant distribution in the arch. Additionally, six implants were enough to support mandibular implant fixed prosthesis due to favorable bone quality in mandible (types I-II) as apposed to maxilla (types III-IV). The mandibular definitive prosthesis was planned to be splinted using precision attachments. At this stage preventive maintenance of the abutment teeth for the temporary prosthesis was, also, performed.

**Figure 6:**
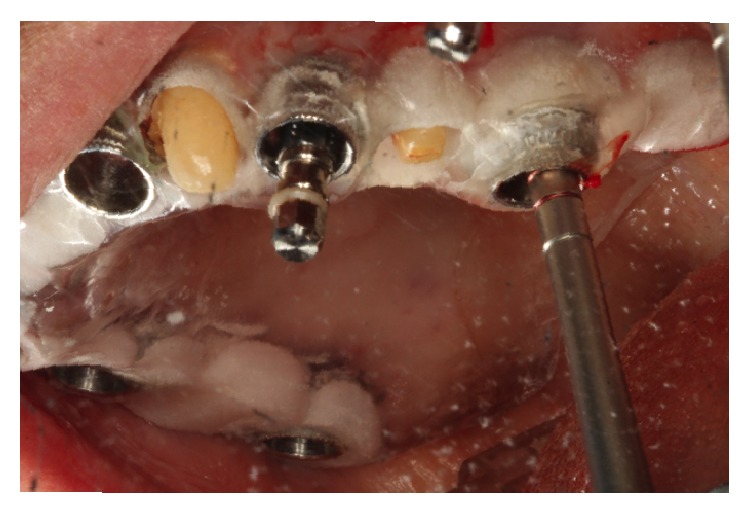
Internal sinus lift and implant osteotomy prepared through the surgical guide for site # 14.

Staging the case allowed for a novel technique for sinus lift procedures through a surgical guide using Salvin/Drew osteotomes (Figure 6).

The CBCT evaluation revealed 7.0 mm of residual bone in areas 3 and 14. Ideal implant placement indicated bilateral sinus lifts at the areas of 3 and 14 and simultaneous ridge expansion through the surgical guide, utilizing the Salvin osteotomes # 2.6, 3.1, and 3.8 mm and the intermediary Salvin/Drew osteotomes [10] 2.6 and 3.1 mm. The intermediary osteotomes were tapped through cortical crestal bone to ensure that the Salvin osteotomes engaged the site. It should be noted that as soon as Salvin/Drew osteotomes reach the initial hub, the next Salvin osteotome can be engaged. The Salvin osteotome # 3.8 mm was then tapped to the final length of 1 mm from the sinus floor. To ensure the final length is accurate, the operator needs to consider the height of the soft tissue and the height of the sleeve in the surgical guide, through which the osteotome is inserted. For site 14, planning on the software revealed 7.0 mm of residual ridge, 5.0 mm in height of the guide sleeve, and the 6.0 mm in soft tissue height. The final length on the last osteotome of 17.0 mm was marked with a stop cylinder, when used through the surgical guide. The guide could now be easily removed. The osteotomy was filled with bone graft material (Bio-Oss, Osteohealth) and osteotome # 3.8 mm was pushed 1.0 mm from the floor of the sinus. The authors recommend using the osteotome with particulate graft material, when the concave tip of the osteotome is 1 mm coronal to the sinus floor. Using this protocol, the graft material, and not the osteotome, is elevating the sinus [11]. The addition of bone graft material was repeated 4 times until the desired length of 17.0 mm was achieved, resulting in a 4 mm internal lift.

**Figure 7:**
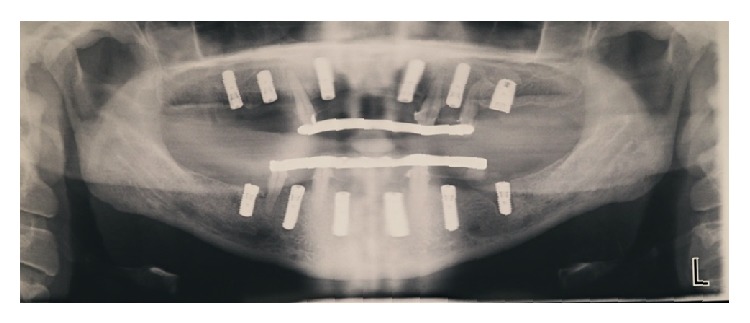
Panoramic radiograph shows the placement of six implants per arch. Note metal-reinforced fixed provisional prostheses retained by retained teeth.

For the prepared osteotomy at site # 14 an SLActive 4.8 × 10 mm was planned to be placed, but due to lack of initial stability a 6 × 10 mm MTX microtextured surface implant was eventually placed. Utilizing the same technique for site # 3 we achieved an increase of 5.0 mm in height which allowed us to place as planned a 4.1 mm × 12.0 mm SLActive implant. Utilizing a tooth-borne surgical guide enabled an easy removal of the guide and radiographic evaluation with 2 periapical X-rays to confirm adequate sinus elevation at sites # 3 and 14. The provisional fixed prostheses were then cemented back, which was an advantage of staging the treatment. The provisional pontic areas above the implants were relieved slightly to allow for minor swelling (Figure 7).

Pre-operative medications for the guided surgery and the simultaneous sinus lifts were prescribed 1 hour before the appointment included 2 grams of amoxicillin [12] and 2 tablets of an Medrol dose pack. Postoperative medications that included amoxicillin 500 mg every 8 hours for 7 days, chlorhexidine gluconate rinse twice a day for 2 weeks, Tylenol # 3 every 4 hours as needed for 7 days, and Medrol dose pack (methylprednisolone 4 mg) were prescribed.

**Figure 8:**
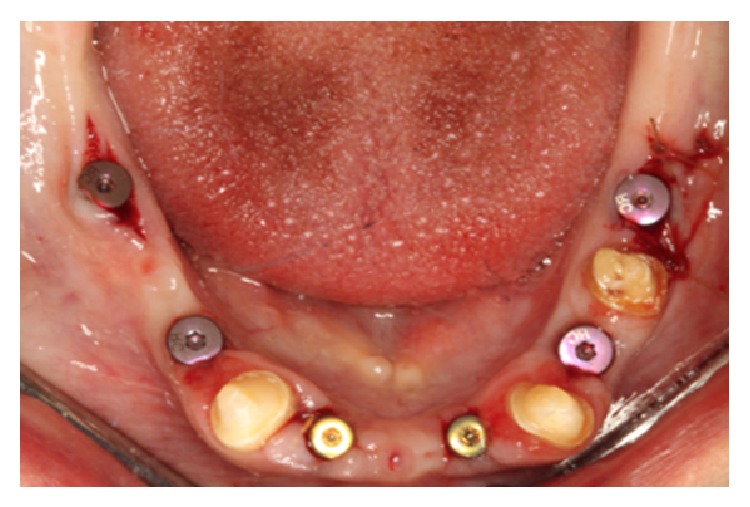
Soft tissue enhancement around lower molar implants, during stage 2 implant uncovery.

**Figure 9:**
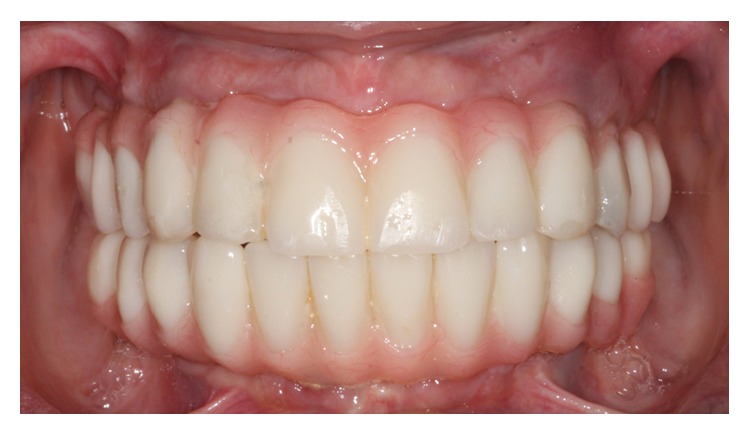
Intraoral photograph shows screw-retained implant provisional prostheses. Pink acrylic was added to enhance esthetics, which will be used as “blue-print” for the definitive prostheses.

After 3 months, all the implants were uncovered and periodontal plastic surgery, with partial thickness flaps, 2 vertical releasing incisions, and an apically positioned flap, was performed for site 3 and the mandibular molar areas due to lack of attached gingiva (Figure 8). This inadequate attached gingiva is common postextraction/GBR, where the mucogingival junction is advanced coronally to achieve closure. Implant level impressions were made for maxillary and mandibular implants which were then poured and mounted against the previous mounted casts of provisional prostheses. Screw-retained provisional prostheses were fabricated with apical pink acrylic to avoid excessively long nonesthetic prostheses. The abutment teeth # 13, 20, 22, 27, and 29 were extracted and augmented with mineralized crushed cortical bone (RegenerOss Allograft, Biomet 3i) and a resorbable collagen membrane (OsseoGuard Flex, Biomet 3i). The screw-retained provisional prostheses were delivered at this point (Figure 9).

**Figure 10:**
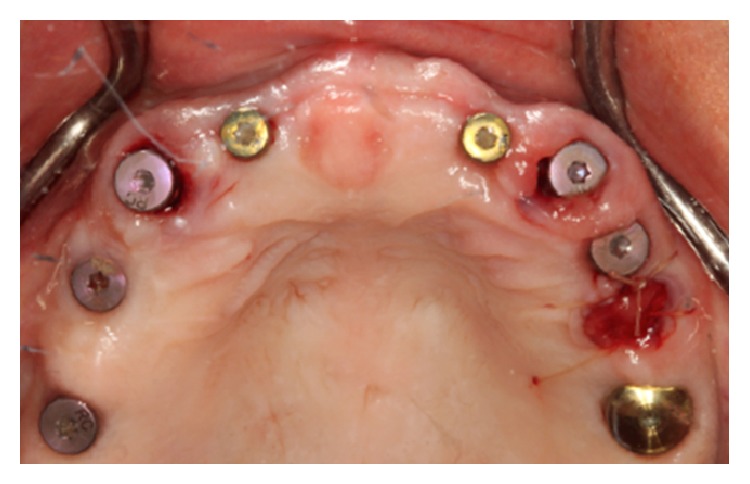
Flapless immediate one-stage implant placement at # 6 and # 11 extraction sockets.

Additionally, flapless immediate one-stage bone level SLActive implants were placed at # 6 and # 11 extraction sockets, through the surgical guide. Peri-implant augmentation with mineralized crushed cortical bone, prior to healing abutment placement, was performed. The sites were stabilized with Vicryl 4-0 sutures after easy removal of the mucosal-tooth-borne guide (Figure 10).

**Figure 11:**
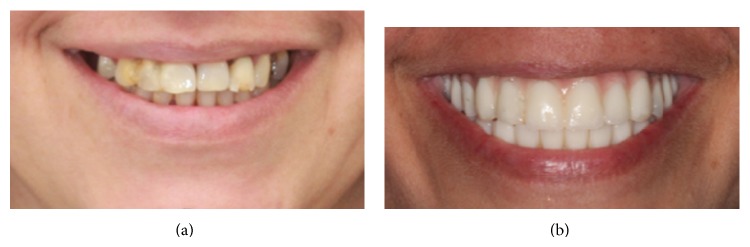
Photographs show patient's smile. (a) Pre-treatment. (b) During treatment.

**Figure 12:**
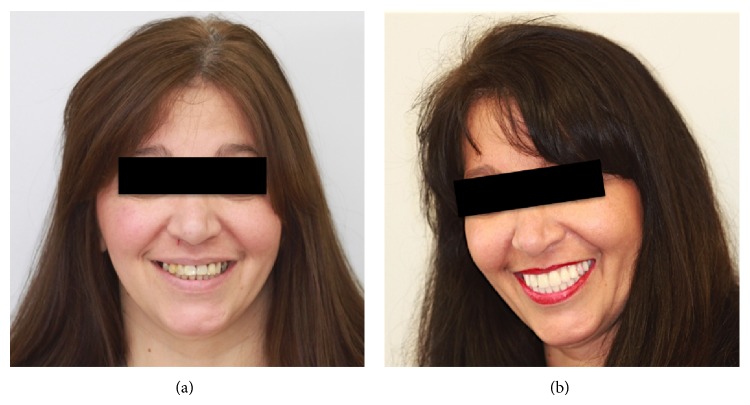
Frontal views for patient's smile. (a) Pre-treatment. (b) During treatment.

Screw-retained prostheses were hollowed in positions # 6 and 11 and temporary cylinders were attached to the immediately placed one-stage implants # 6 and 11. The cylinders were picked up intraorally using temporary acrylic material (Alike, GC America Inc., Alsip, IL). The provisional fixed prostheses were finished and polished around newly added temporary cylinders and delivered back to the patient (Figures 11 and 12).

Six weeks allowed for healing around immediately placed implants # 6 and 11. Fixture level impressions were taken for maxillary and mandibular implants and poured in dental stone (Silky Rock, Whip mix) for master casts. The master casts were then cross-mounted against the duplicate cast of corrected implant retained provisional prostheses. The design of the definitive prostheses involved screw-retained porcelain fused to metal for ease of retrievability when needed on follow-up visits. The use of cast metal frame, which is an integral part of the definitive prosthesis, allows predictable sectioning and soldering in case of misfit upon try-in stage and provides high strength while giving enough space for porcelain to get an excellent esthetic outcome. The maxillary and mandibular definitive prostheses were designed in 3 separate sections in each arch. The provisional and the definitive prostheses were fabricated with even posterior teeth contacts in centric jaw position, anterior guidance in protrusion, and canine guidance with immediate posterior disocclusion on lateral eccentric movements. Since 6 implants were placed for the mandible, the definitive prostheses had precision attachments on the distal aspects of the mandibular canines. Designing the prostheses with multiple sections allows for easier maintenance and management of future prosthetic complications. Post-insertion management includes delivery of night guard and follow-up visits every 3-4 months for the first year and then every 6 months thereafter.

## 3. Results and Discussion

Prosthetic rehabilitation in the course of periodontal, surgical intervention is very difficult for both the patient and the clinicians. The four basic techniques for transition from a questionable or hopeless dentition to a fixed reconstruction are removable dentures, transitional mini-implants, immediately loaded restorations, and the staged approach [13]. The advantages of a staged approach are numerous.

Absence of premature loading and presence of balanced occlusion due to adequate fixed restorations allowed for space maintenance with GBR and tenting screw techniques and uneventful bone and soft tissue augmentation of the maxilla and the mandible, without any mechanical overload.

The second set of the provisional restorations also contributed to tissue sculpting of the edentulous pontic areas.

The modified osteotome technique, through the CBCT predesigned mucosa-teeth supported surgical guide, with simultaneous implant placement, ensures an accurate position for the internal sinus lifts and, respectively, the implant placements on the bone crest, when a flapless approach is performed. As the Salvin/Drews osteotomes are stepped up in size, the tapping or pushing should be done in a slow, gentle manner to allow for bone expansion, atraumatic sinus elevation, and simultaneous preparation of the implant osteotomy, through the guide. The fixed restorations prevented mechanical load on the tenting screws and internal sinus lifts sites, which healed uneventfully, with an increase in bone height of 4 to 5 mm bilaterally. The immediately placed maxillary molar implants were successfully osseointegrated.

Following the use of CBCT scan for diagnosis and treatment planning, 3 types of surgical guides can be fabricated based on the anatomical structure used for support upon surgery (i.e., bone, teeth, or mucosa). The selection of the type of CBCT scan surgical guide depends on surgeon preference, the proposed plan for implant surgery, and whether or not a full thickness mucoperiosteal flap is needed for direct access to alveolar bone. Ozan et al. [14] reported that the use of 3 types of surgical guides results in angular deviation of 4.1° ± 2.3° and linear deviation of 1.1 ± 0.7 mm at the implant neck and 1.41 ± 0.9 mm at the implant apex away from the proposed implant site [14]. The same research group found that the use of tooth supported surgical guide results in 50% reduction in angular deviation when compared to the other two types. The surgical guide that was used in our report for implants placements is classified as mucosa supported; however the remaining dentition helped for orientation and stability of the guide for optimum atraumatic implant placement. Additionally, the design of this guide allows easy removal to clean the surgical site, in areas where there is tissue remaining after punch incisions at the beginning of the site preparation when a flapless approach is performed.

The advantages of staging the patient with provisional fixed prostheses during all phases of treatment are numerous. The staged approach gave the operators the opportunity to increase the keratinized tissue with apically positioned split thickness flaps and palatal roll procedures [15] on the second stage at implant uncovery.

It provides patients with superior esthetics function and comfort while avoiding excessive loading over grafted or second stage implants sites. The stress of the procedure is minimized to both the patient and the surgeon, since the transitional prosthesis can be easily adjusted to achieve ideal soft tissue contours, while the patient is wearing the provisional restoration. Additionally, the patient was given the time to adjust to the new prosthesis and perform excellent oral hygiene, before the cementation of the final restoration. Physical and psychological comfort of the patient was of major importance.

Alternatives to staging the case are removable temporary prosthesis option or immediate loading. If insertion torque is less than 35 ncm, the patient should still have to wear a removable prosthesis. Failures have been reported in the literature due to mechanical overload of the overlying prosthesis and micromovement of the fixtures [16].

Alternative to a mucosa-tooth supported guide is a nontooth supported radiographic surgical guide. Disadvantages included further fixation of this type of guide to the buccal bone and less access to the surgical field for the clinician.

Risks through the course of treatment include inadequate oral hygiene and unpredictable change in the medical history of the patient. In our case, the patient was diagnosed with stomach ulcers at early stages of treatment and ibuprofen was altered to Tylenol. The patient was sent back to the physician for an INR.

Other complications include fractures of the temporary prosthesis due to excessive mastication forces. In cases of bruxism, the patient should additionally be provided with a night guard to minimize the risk of mechanical complications [17].

## 4. Conclusion

Although the staged approach requires more time and effort in planning the case and adequate knowledge of three-dimensional prosthetic guided treatment planning, it is suggested that it is considered as an option for full mouth rehabilitation of patients. Staging the case, fixed provisional restorations, and the fabrication of a tooth supported radiographic/surgical guide as part of the treatment for these patients are recommended when possible. These steps add to patient satisfaction and success of the prosthetic directed implant placement and fixed restoration.
